# Hydrogen Sulfide Reduces Ischemia and Reperfusion Injury in Neuronal Cells in a Dose- and Time-Dependent Manner

**DOI:** 10.3390/ijms221810099

**Published:** 2021-09-18

**Authors:** Stefanie Scheid, Max Goeller, Wolfgang Baar, Jakob Wollborn, Hartmut Buerkle, Günther Schlunck, Wolf Lagrèze, Ulrich Goebel, Felix Ulbrich

**Affiliations:** 1 Department of Anesthesiology and Critical Care, Medical Center-University of Freiburg, Faculty of Medicine, University of Freiburg, 79106 Freiburg, Germany; stefanie.scheid@uniklinik-freiburg.de (S.S.); max.goeller@uniklinik-freiburg.de (M.G.); wolfgang.baar@uniklinik-freiburg.de (W.B.); jwollborn@bwh.harvard.edu (J.W.); hartmut.buerkle@uniklinik-freiburg.de (H.B.); 2 Department of Anesthesiology, Perioperative and Pain Medicine, Brigham and Woman’s Hospital, Harvard Medical School, Boston, MA 02115, USA; 3 Eye-Center, Medical Center-University of Freiburg, Faculty of Medicine, University of Freiburg, 79106 Freiburg, Germany; guenther.schlunck@uniklinik-freiburg.de (G.S.); wolf.lagreze@uniklinik-freiburg.de (W.L.); 4 Department of Anesthesiology and Critical Care Medicine, St. Franziskus-Hospital, 48145 Muenster, Germany; ulrich.goebel@uniklinik-freiburg.de

**Keywords:** hydrogen sulfide, H_2_S, ischemia–reperfusion injury, neuroprotection, retinal ganglion cells

## Abstract

Background: The ischemia-reperfusion injury (IRI) of neuronal tissue, such as the brain and retina, leads to possible cell death and loss of function. Current treatment options are limited, but preliminary observations suggest a protective effect of hydrogen sulfide (H_2_S). However, the dosage, timing, and mechanism of inhaled H_2_S treatment after IRI requires further exploration. Methods: We investigated possible neuroprotective effects of inhaled H_2_S by inducing retinal ischemia–reperfusion injury in rats for the duration of 1 h (120 mmHg), followed by the administration of hydrogen sulfide (H_2_S) for 1 h at different time points (0, 1.5, and 3 h after the initiation of reperfusion) and at different H_2_S concentrations (120, 80, and 40 ppm). We quantified the H_2_S effect by conducting retinal ganglion cell counts in fluorogold-labeled animals 7 days after IRI. The retinal tissue was harvested after 24 h for molecular analysis, including qPCR and Western blotting. Apoptotic and inflammatory mediators, transcription factors, and markers for oxidative stress were investigated. Histological analyses of the retina and the detection of inflammatory cytokines in serum assays were also performed. Results: The effects of inhaled H_2_S were most evident at a concentration of 80 ppm administered 1.5 h after IRI. H_2_S treatment increased the expression of anti-apoptotic Bcl-2, decreased pro-apoptotic Bax expression, reduced the release of the inflammatory cytokines IL-1β and TNF-α, attenuated NF-κB p65, and enhanced Akt phosphorylation. H_2_S also downregulated NOX4 and cystathionine β-synthase. Histological analyses illustrated a reduction in TNF-α in retinal ganglion cells and lower serum levels of TNF-α in H_2_S-treated animals after IRI. Conclusion: After neuronal IRI, H_2_S mediates neuroprotection in a time- and dose-dependent manner. The H_2_S treatment modulated transcription factor NF-κB activation and reduced retinal inflammation.

## 1. Introduction

Diseases associated with ischemia–reperfusion injury (IRI) of the central nervous system lead to neuronal cell death. Well-known examples of this disease group are traumatic brain injury and stroke, but diseases of the eye, such as retinal vascular occlusion, diabetic retinopathy, and glaucoma, are also associated with IRI. The retinal ganglion cells are very vulnerable to ischemia and have little potential for regeneration following injury. As in other neuronal cells, the IRI of retinal ganglion cells activates several apoptotic and inflammatory signaling pathways that ultimately trigger cell death [1]. 

A possible treatment that could suppress this inevitable neuronal cell death is the exposure to hydrogen sulfide (H_2_S). Although H_2_S is traditionally considered a toxic gas, it also functions as a natural endogenous gasotransmitter in normal physiological processes. It plays an important functional role in the central nervous system, where it appears to be predominantly synthesized by the enzyme cystathionine beta-synthase (CBS), which has been localized in astrocytes, as well as in microglia and neurons [2,3]. H_2_S is normally stored as bound sulfane sulfur and is released by excitatory stimulation [4]. Elevated levels of endogenously produced H_2_S are detected after middle cerebral artery occlusion and may contribute to the pathology of stroke [5]. 

At low doses, exogenously applied H_2_S may be beneficial in pathophysiological processes. Several in vitro and in vivo studies have demonstrated that H_2_S may contribute to neuroprotective effects. This is likely mediated through a variety of mechanisms, including vasodilatation via the activation of potassium-dependent ATP channels [6], direct endothelial vasorelaxation [7], antioxidation by radical scavenging [8], interference with intracellular signal transduction, and a consecutive reduction in inflammation [9,10]. However, at high concentrations, H_2_S causes toxic effects, probably by inhibiting cytochrome c oxidase and blocking aerobic ATP generation, leading to subsequent cell death [11].

Only a few studies have examined the use of inhaled H_2_S as a treatment for neuronal injury. Geng et al. investigated the impact of inhaled H_2_S after cardiac arrest and cardiopulmonary resuscitation in rats. The application of 80 ppm H_2_S immediately after injury reduced the permeability of the blood–brain barrier, decreased brain edema, attenuated neurological dysfunction, and increased survival [12]. Li et al. also used a cardiac arrest and resuscitation model to confirm the protective effects of H_2_S and showed similar anti-inflammatory potency of both 80 and 40 ppm H_2_S [13]. Evidence of an H_2_S-associated neuroprotection of neuronal cells in the eye was shown by Biermann et al., who demonstrated that 80 ppm H_2_S administered immediately before IRI decreased retinal ganglion cell death and reduced retinal inflammation [14]. 

To our knowledge, the effects of various concentrations of H_2_S administered at different time points after neuronal IRI have not been studied so far. Therefore, we performed investigations in a rat model of retinal IRI to determine the most effective inhaled H_2_S treatment as indicated by various apoptotic and inflammatory markers.

## 2. Results

### 2.1. H_2_S Inhalation Protects Retinal Ganglion Cells from IRI-Induced Apoptosis at Specific Concentrations and Times

**Figure 1:**
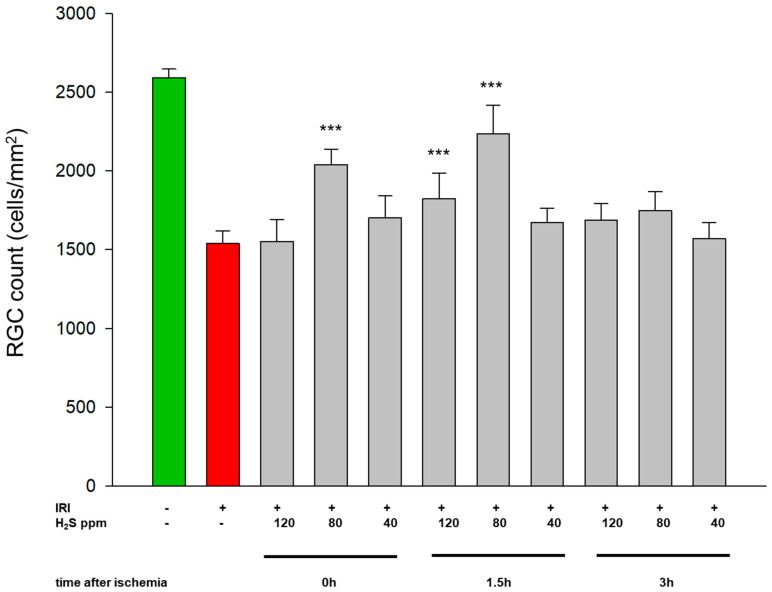
Time- and dose-dependent influence of H_2_S inhalation on retinal ganglion cell count after ischemia–reperfusion injury (IRI). Quantification of retinal ganglion cell density (cells/mm^2^, data are the mean ± SD, *n* = 8, *** = *p* < 0.001; IRI vs. IRI + 80 ppm H_2_S at 0 h and 1.5 h, vs. IRI + 120 ppm H_2_S at 1.5 h).

**Figure 2:**
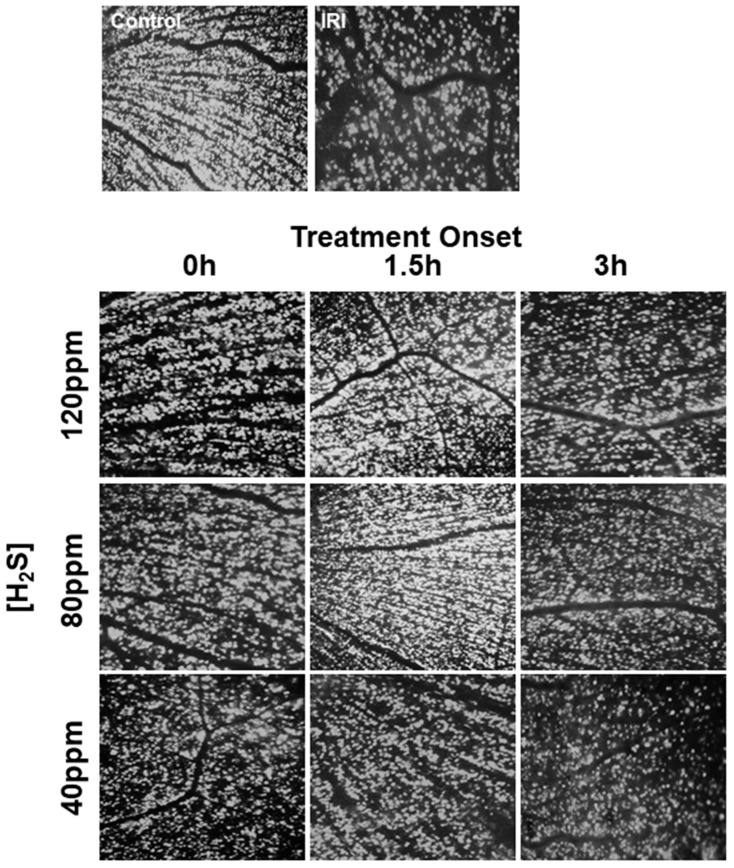
Time- and dose-dependent influence of H_2_S inhalation on vital retinal ganglion cells after ischemia–reperfusion injury (IRI). Representative flat mount images (*n* = 8) of fluorogold-labeled retinal ganglion cells 7 days after IRI and immediate (0 h) and delayed (i.e., 1.5 and 3 h) H_2_S inhalation (40, 80 and 120 ppm).

IRI induced a loss of approximately 50% of the retinal ganglion cells (IRI: 1540 ± 78 vs. untreated: 2592 ± 54; Figure 1, Col. 1 and 2). Inhaled H_2_S was able to attenuate this effect at concentrations of 120 and 80 ppm at specific time points (Figure 1, Col 4, 6, 7). At a concentration of 120 ppm, only the inhalation administered at 1.5 h after IRI had a protective effect, as indicated by a significantly reduced cell death (IRI + 120 ppm H_2_S at 1.5 h: 2039 ± 99, *p* < 0.001). The immediate inhalation (time point of 0 h), or inhalation after a time delay of 3 h, had no protective benefit (IRI + 120 ppm H_2_S at 0 h: 1540 ± 78; IRI + 120 ppm H_2_S at 3 h: 1686 ± 105). The administration of 80 ppm H_2_S had a strong protective effect at two time points (0 and 1.5 h), with the best results occurring after inhalation with a 1.5 h delay (IRI + 80 ppm H_2_S at 0 h: 2039 ± 99 and IRI + 80 ppm H_2_S at 1.5 h: 2234 ± 181; both *p* < 0.001). However, no protection against cell death was observed after a time delay of 3 h following IRI. The administration of 40 ppm H_2_S also failed to prevent retinal ganglion cell death at any time point after IRI (IRI + 40 ppm H_2_S at 0 h: 1702 ± 140; IRI + 40 ppm H_2_S at 1.5 h: 1671 ± 92; IRI + 40 ppm H_2_S at 3 h: 1569 ± 103). Representative images of vital retinal ganglion cells and the induction of cell death by IRI, as well as the protective effects of hydrogen sulfide, are shown in Figure 2.

### 2.2. H_2_S Inhalation Reduced Bax Expression and Increased Bcl-2 Expression

**Figure 3:**
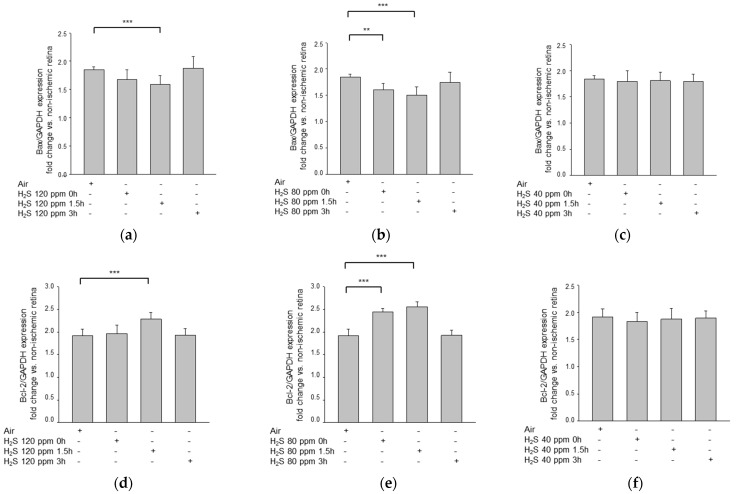
Effect of H_2_S inhalation on retinal expression of Bax and Bcl-2 mRNA. (**a**–**c**) Fold induction of Bax mRNA expression in ischemic retinal tissue compared to GAPDH in relation to the corresponding non-ischemic retinae analyzed by RT-PCR after (**a**) 120 ppm H_2_S inhalation (data are mean ± SD, *n* = 8; ** = *p* < 0.01, IRI vs. 120 ppm H_2_S at 1.5 h), (**b**) 80 ppm H_2_S inhalation (data are mean ± SD, *n* = 8; *** = *p* < 0.001, IRI vs. IRI + 80 ppm H_2_S at 1.5 h; ** = *p* < 0.01, IRI vs. IRI + 80 ppm H_2_S at 0 h), and (**c**) 40 ppm H_2_S inhalation. (**d**–**f**) Fold induction of Bcl-2 mRNA expression in ischemic retinal tissue compared to GAPDH in relation to the corresponding non-ischemic retinae analyzed by RT-PCR after (**d**) 120 ppm H_2_S inhalation (data are mean ± SD, *n* = 8; *** = *p* < 0.001, IRI vs. 120 ppm H_2_S at 1.5 h), (**e**) 80 ppm H_2_S inhalation (data are mean ± SD, *n* = 8; *** = *p* < 0.001, IRI vs. IRI + 80 ppm H_2_S at 1.5 h; *** = *p* < 0.001, IRI vs. IRI + 80 ppm H_2_S at 0 h) and (**f**) 40 ppm H_2_S inhalation.

H_2_S also affected the expression of pro-apoptotic Bax and anti-apoptotic Bcl-2 after IRI (Figure 3). The strongest effect in each case was observed for the 80 ppm treatment applied with a delay of 1.5 h (Bax—IRI: 1.84 ± 0.06 vs. IRI + 80 ppm H_2_S at 1.5 h: 1.50 ± 0.16, *p* < 0.001; Bcl-2—IRI: 0.48 ± 0.04 vs. IRI + 80 ppm H_2_S at 1.5 h: 1.28 ± 0.05, *p* < 0.001). However, the immediate application of 80 ppm H_2_S also significantly reduced Bax and increased Bcl-2 expression (Bax—IRI: 1.84 ± 0.06 vs. IRI + 80 ppm H_2_S at 0 h: 1.60 ± 0.12, *p* < 0.01; Bcl-2—IRI: 0.48 ± 0.04 vs. IRI + 80 ppm H_2_S at 0 h: 1.22 ± 0.04, *p* < 0.001). By contrast, the effect was attenuated but still statistically significant following treatment with 120 ppm H_2_S with a delay of 1.5 h (Bax—IRI: 1.84 ± 0.06 vs. IRI + 120 ppm H_2_S at 1.5 h: 1.59 ± 0.16, *p* < 0.01; Bcl-2—IRI: 0.48 ± 0.04 vs. IRI + 120 ppm H_2_S at 1.5 h: 1.14 ± 0.07, *p* < 0.001), whereas the immediate application of 120 ppm H_2_S had no effect. A delay of 3 h, as well as treatment with 40 ppm H_2_S, had no influence on Bax or Bcl-2 expression. 

### 2.3. H_2_S Inhalation Reduced NOX4 Expression

**Figure 4:**
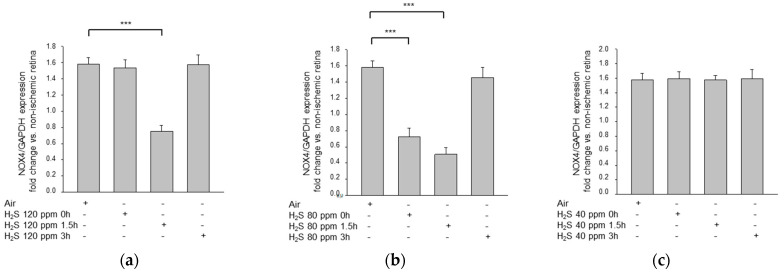
Effect of H_2_S inhalation on retinal expression of NOX-4 mRNA. Fold induction of NOX-4 mRNA expression in ischemic retinal tissue compared to GAPDH in relation to the corresponding non-ischemic retinae analyzed by RT-PCR after (**a**) 120 ppm H_2_S inhalation (data are mean ± SD, *n* = 8; *** = *p* < 0.001, IRI vs. 120 ppm H_2_S at 1.5 h), (**b**) 80 ppm H_2_S inhalation (data are mean ± SD, *n* = 8; *** = *p* < 0.001, IRI vs. IRI + 80 ppm H_2_S at 0 h and vs. IRI + 80 ppm H_2_S at 1.5 h), and (**c**) 40 ppm H_2_S inhalation.

H_2_S treatment inhibited the expression of NOX4, a member of the NADPH oxygenase family that is critically involved in the production of reactive oxygen species (Figure 4). The most potent treatment was 80 ppm with a 1.5 h delay (IRI: 1.58 ± 0.08 vs. IRI + 80 ppm H_2_S at 1.5 h: 0.51 ± 0.08, *p* < 0.001, IRI + 80 ppm H_2_S at 0 h: 0.73 ± 0.10, *p* < 0.001; IRI + 120 ppm H_2_S at 1.5 h: 0.75 ± 0.07, *p* < 0.001).

### 2.4. H_2_S Inhalation Decreased Inflammatory Cytokine Expression

**Figure 5:**
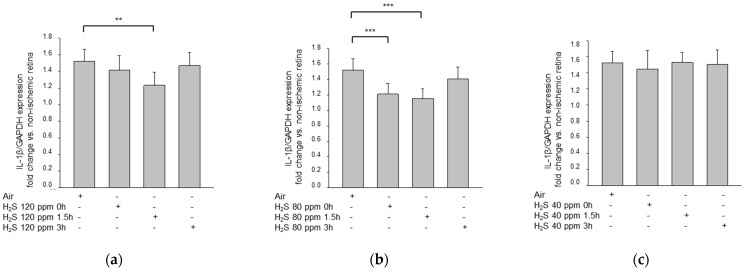

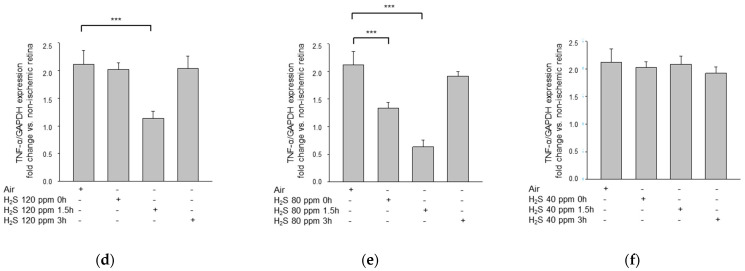
Effect of H_2_S inhalation on the retinal expression of IL-1ß and TNF-α mRNA. (**a**–**c**) Fold induction of IL-1ß mRNA expression in ischemic retinal tissue compared to GAPDH in relation to the corresponding non-ischemic retinae analyzed by RT-PCR after (**a**) 120 ppm H_2_S inhalation (data are mean ± SD, *n* = 8; ** = *p* ≤ 0.01, IRI vs. 120 ppm H_2_S at 1.5 h), (**b**) 80 ppm H_2_S inhalation (data are mean ± SD, *n* = 8; *** = *p* < 0.001 IRI vs. IRI + 80 ppm H_2_S at 0 h and vs. IRI + 80 ppm H_2_S at 1.5 h), and (**c**) 40 ppm H_2_S inhalation. (**d**–**f**) Fold induction of TNF-α mRNA expression in ischemic retinal tissue compared to GAPDH in relation to the corresponding non-ischemic retinae analyzed by RT-PCR after (**d**) 120 ppm H_2_S inhalation (data are mean ± SD, *n* = 8; *** = *p* < 0.001, IRI vs. 120 ppm H_2_S at 1.5 h), (**e**) 80 ppm H_2_S inhalation (data are mean ± SD, *n* = 8; *** = *p* < 0.001, IRI vs. IRI + 80 ppm H_2_S at 0 h and vs. IRI + 80 ppm H_2_S at 1.5 h), and (**f**) 40 ppm H_2_S inhalation.

H_2_S treatment decreased the pro-inflammatory cytokine response, as indicated by the decreased expression of the inflammatory cytokines IL-1β and TNF-α, as determined by qPCR (Figure 5). The extent of the downregulation depended on the specific concentration and timing of the H_2_S treatment. Significant changes were noted at 80 ppm at 1.5 h and 0 h, and at 120 ppm at 1.5 h (IL-1ß—IRI: 2.12 ± 0.24 vs. IRI + 80 ppm H_2_S at 1.5 h: 0.64 ± 0.12, *p* < 0.001; IRI + 80 ppm H_2_S at 0 h: 1.33 ± 0.10, *p* < 0.001; IRI + 120 ppm H_2_S at 1.5 h: 1.14 ± 0.13, *p* < 0.01; TNF-α—IRI: 1.52 ± 0.15 vs. IRI + 80 ppm H_2_S at 1.5 h: 1.15 ± 0.13, *p* < 0.001, IRI + 80 ppm H_2_S at 0 h: 1.21 ± 0.14, *p* < 0.001; IRI + 120 ppm H_2_S at 1.5 h: 1.23 ± 0.15, *p* < 0.01). Treatment at the other time points, as well as the 40 ppm H_2_S treatment, had no effect on cytokine expression.

### 2.5. H_2_S Inhalation Inhibits TNF-α Expression in the Ganglion Cell Layer

**Figure 6:**
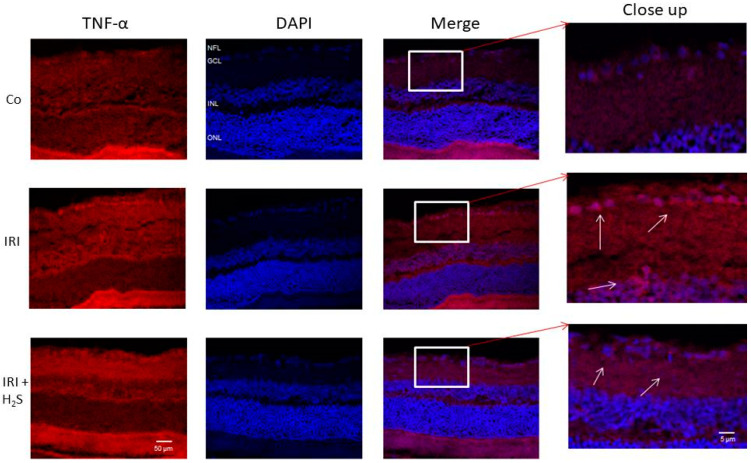
TNF-α expression after unilateral IRI and H_2_S inhalation. Double staining was performed to clarify in which areas of the retina the expression of TNF-α occurs. Cross-sections of the retinae 7 days after unilateral IRI showed that IRI led to an increased expression of TNF-α mainly in the retinal ganglion cell layer, as well as in the inner nuclear layer. After the application of H_2_S, the expression was attenuated. Abbreviations: NFL  = nerve fiber layer; GCL  = ganglion cell layer; INL  = inner nuclear layer; ONL  = outer nuclear layer.

The cytokine TNF-α is a key regulator of the inflammatory response among others. TNF-α was detected mainly in the inner nuclear layer and the area of the ganglion cell layer in retinal cross-sections of IRI eyes (Figure 6). H_2_S treatment (80 ppm, time interval 1.5 h) resulted in weaker staining, indicating decreased TNF-α expression.

### 2.6. H_2_S Inhalation Attenuates Inflammatory Cytokine Expression in Peripheral Blood

**Figure 7:**
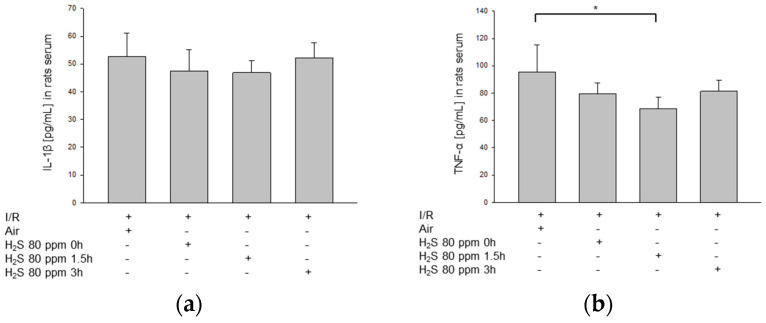
Effect of H_2_S inhalation on IL-1β and TNF-α levels in peripheral blood samples. (**a**) Analysis of IL-1β levels after IRI and subsequent H_2_S inhalation (80 ppm) (pg/mL). (**b**) Analysis of TNF-α levels after IRI and subsequent H_2_S inhalation (80 ppm) (pg/mL; data are mean ± SD, *n* = 8; * = *p* < 0.05, IRI vs. IRI + 80 ppm H_2_S at 1.5 h).

H_2_S treatment decreased TNF-α levels in the peripheral blood when compared to room air treatment after retinal IRI (IRI: 95 ± 20 pg/µL; Figure 7b). However, the decrease was not statistically significant after immediate treatment or after treatment with a delay of 3 h (IRI + 80 ppm H_2_S at 0 h: 79 ± 8 pg/µL; IRI + 80 ppm H_2_S at 3 h: 81 ± 8 pg/µL). A more pronounced effect was detected when the H_2_S treatment was given at 1.5 h after IRI (IRI + 80 ppm H_2_S at 1.5 h: 68 ± 8 pg/µL, *p* < 0.05). In contrast to TNF-α, only a slight decrease in IL-1β was detectable in serum after immediate and delayed (1.5 h) H_2_S administration, which was not significant (Figure 7a; IRI: 53 ± 8 pg/µL vs. IRI + 80 ppm H_2_S at 0 h: 47 ± 8 pg/µL and IRI + 80 ppm H_2_S at 1.5 h: 47 ± 4 pg/µL). With a 3 h delay, no difference in IL-1β levels was detectable (IRI: 53 ± 8 pg/µL vs. IRI + 80 ppm H_2_S at 0 h: 52 ± 5 pg/µL).

### 2.7. H_2_S Inhalation Decreased CBS Expression

**Figure 8:**
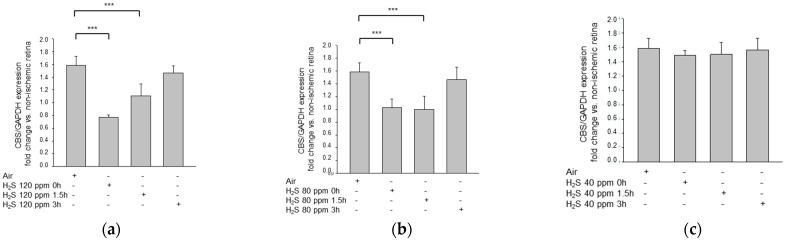
Effect of H_2_S inhalation on the retinal expression of CBS mRNA. Fold induction of CBS mRNA expression in ischemic retinal tissue compared to GAPDH in relation to the corresponding non-ischemic retinae analyzed by RT-PCR after (**a**) 120 ppm H_2_S inhalation (data are mean ± SD, *n* = 8; *** = *p* < 0.001, IRI vs. 120 ppm H_2_S at 0 h and vs. 120 ppm H_2_S at 1.5 h), (**b**) 80 ppm H_2_S inhalation (data are mean ± SD, *n* = 8; *** = *p* < 0.001, IRI vs. IRI + 80 ppm H_2_S at 0 h and vs. IRI + 80 ppm H_2_S at 1.5 h), and (**c**) 40 ppm H_2_S inhalation.

The endogenous production of H_2_S is linked to the activity of enzymes, such as CBS. The 120 ppm H_2_S treatment applied immediately after reperfusion showed the strongest inhibition of CBS expression (Figure 8; IRI: 1.58 ± 0.14 vs. IRI + 120 ppm H_2_S at 0 h: 0.77 ± 0.04, *p* < 0.001). However, a delayed application (1.5 h) of 120 ppm H_2_S, as well as the 80 ppm treatments at 0 h and 1.5 h, also caused a significant reduction in CBS expression (IRI: 1.58 ± 0.14 vs. IRI + 120 ppm H_2_S at 1.5 h: 1.11 ± 0.18, *p* < 0.001; IRI + 80 ppm H_2_S at 0 h: 1.03 ± 0.13, *p* < 0.001; IRI + 80 ppm H_2_S at 1.5 h: 1.00 ± 0.20, *p* < 0.001). 

### 2.8. H_2_S Inhalation Reduces NF-κB Phosphorylation 

**Figure 9:**
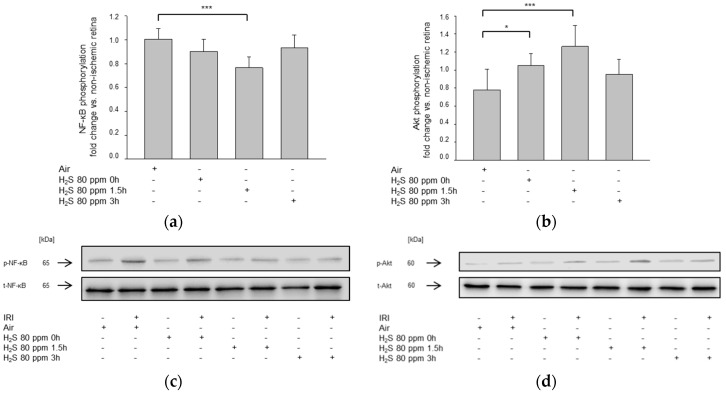
(**a**,**c**) Effect of H_2_S inhalation on the retinal expression of NF-κB phosphorylation. Densitometric analysis of *n* = 8 Western blots for NF-κB phosphorylation after time-dependent inhalation of 80 ppm H_2_S (data are mean ± SD, *n* = 8; *** = *p* < 0.001, IRI vs. IRI + 80 ppm H_2_S at 1.5 h) and representative Western blot image (*n* = 8) showing the suppression of retinal phosphorylation of NF-κB compared to total NF-κB. (**b**,**d**) Effect of H_2_S inhalation on the retinal expression of Akt phosphorylation. Densitometric analysis of *n* = 8 Western blots for Akt phosphorylation after time-dependent inhalation of 80 ppm H_2_S (data are mean ± SD, *n* = 8; *** = *p* < 0.001, IRI vs. IRI + 80 ppm H_2_S at 1.5 h, and * = *p* < 0.05, IRI vs. IRI + 80 ppm H_2_S at 0 h) and representative Western blot image (*n* = 8) showing the increase of retinal phosphorylation of Akt compared to total Akt.

We also examined the activation of the transcription factor NF-κB, which plays a critical role in inflammatory and apoptotic processes (Figure 9a). We used 80 ppm H_2_S, as this showed to have the best protective effect on retinal ganglion cells. Inhalation immediately and 3 h after retinal IRI only slightly reduced NF-κB phosphorylation compared to IRI without H_2_S treatment (IRI: 1.00 ± 0.09 vs. IRI + 80 ppm H_2_S at 0 h: 0.90 ± 0.10 and IRI + 80 ppm H_2_S at 3 h: 0.93 ± 0.11, n.s.). However, inhalation 1.5 h after IRI reduced NF-κB phosphorylation significantly (IRI: 1.00 ± 0.09 vs. IRI + 80 ppm H_2_S at 1.5 h: 0.77 ± 0.09, *p* < 0.05). 

### 2.9. H_2_S Inhalation Induced Akt Phosphorylation 

Western blot analysis showed that immediate treatment with 80 ppm H_2_S enhanced Akt phosphorylation (Figure 9b; IRI: 0.82 ± 0.24 vs. IRI + 80 ppm H_2_S at 0 h: 1.10 ± 0.11, *p* < 0.05), and this effect was even more pronounced when H_2_S was applied with a delay of 1.5 h (IRI + 80 ppm H_2_S at 1.5 h: 1.26 ± 0.23, *p* < 0.001). By contrast, extending the treatment delay time to 3 h caused only a mild and not statistically significant increase (IRI + 80 ppm H_2_S at 3 h: 0.95 ± 0.16, n.s.). 

## 3. Discussion

This study investigated the neuroprotective effect of inhaled H_2_S. The following are the key findings: (1) The highest protective effect of H_2_S was observed with treatment at 80 ppm applied 1.5 h after IRI; (2) H_2_S treatment decreased the expression of the pro-inflammatory cytokines IL-1β and TNF-α; (3) H_2_S treatment reduced the expression of the pro-apoptotic Bax protein, increased the expression of the anti-apoptotic BCL-2, and attenuated NOX4 expression; (4) H_2_S treatment also diminished CBS expression; (5) H_2_S treatment reduced NF-κB phosphorylation and increased Akt phosphorylation.

The results of this study demonstrate that inhaled H_2_S has protective effects after the IRI of the retina, but the extent of neuroprotection depends on the dosage and the time of application. The application of 80 ppm H_2_S 1.5 h after IRI showed the best protection against retinal ganglion cell death. An immediate treatment with 80 ppm H_2_S significantly reduced cell death too, although to a lesser extent than the 80 ppm treatment at 1.5 h. A concentration of 120 ppm H_2_S provided a favorable effect only after a time delay of 1.5 h, whereas treatment at 40 ppm had no effect on ganglion cell survival after retinal IRI at any time point. 

To date, studies on the neuroprotective effects of inhaled H_2_S have been performed predominantly at concentrations of 40 or 80 ppm. For example, Wei et al. administered inhaled H_2_S to rabbits at a concentration of 80 ppm for 1 h immediately after induced cardiac arrest and resuscitation and after the return of spontaneous circulation (ROSC). They found that H_2_S treatment reduced the adverse histopathological changes in the brain, improved neurological function, and improved survival in H_2_S-treated animals compared with a sham group [15]. Another study tested two H_2_S concentrations (40 or 80 ppm) in rats immediately after induced cardiac arrest and subsequent ROSC. Both concentrations showed a neuroprotective effect in terms of reduced neuronal cell loss, improved neurological function, and better overall survival [16]. A series of experiments in rats by Wei et al. showed that middle cerebral artery occlusion for 2 h followed immediately by a 3 h treatment with H_2_S at either concentration at the onset of reperfusion resulted in improved neurologic function and reduced infarct size at 24 h compared to the sham group. Both concentrations showed a similar protective effect. Similarly, Geng and colleagues studied the neuroprotective effect of 80 ppm H_2_S applied with a time delay of 1 h after the return of spontaneous circulation (ROSC) after induced cardiac arrest and cardiopulmonary resuscitation. They also showed sufficient protection and reduced permeability of the blood–brain barrier, decreased cerebral edema, and improved neurological outcome and survival [12]. However, the neuroprotective effect of H_2_S at a concentration of 120 ppm or at other time intervals following IRI has not been studied previously.

In our study, the neuroprotective effect of H_2_S was investigated for the first time in a dose- and time-dependent manner. The best effect was given with 80 ppm, either immediately after IRI, or after a time delay of 1.5 h from the onset of reperfusion. A concentration of 40 ppm seemed too low to protect against retinal ganglion cell death after IRI. Similarly, a time lag of 3 h seemed too long to protect against an early-onset injury mechanism at all concentrations. The reason why no neuroprotective effect was observed for 120 ppm H_2_S administered immediately after the termination of ischemia is unknown. One may speculate that a toxic component of H_2_S might counteract the protective effect, possibly at the mitochondrial level, but this was not investigated in detail in the present study. No further loss of vital retinal ganglion cells due to H_2_S was observed in our animals. 

Our investigation of the underlying neuroprotective mechanism demonstrated that H_2_S treatment increased Akt phosphorylation and reduced the IRI-induced increase in NF-κB phosphorylation, with each response showing a dose- and time-dependent effect of H_2_S. Akt and the transcription factor NF-κB are significantly involved in anti-apoptotic and inflammatory processes in neuronal IRI [17]. Hypoxia resulting from IRI leads to the activation of Akt, which triggers effects such as pro-survival functions and the inhibition of apoptotic signaling pathways in mitochondria [18,19]. Intracellular signaling triggers an apoptotic stimulus that causes the oligomerization of the Bcl-2 antagonist and Bax, thereby inactivating Bcl-2. Activated Bax then alters the mitochondrial membrane potential, stimulates the release of cytochrome c, and activates caspases [20,21]. 

Another key regulator in neuronal ischemia is nuclear factor kappa B (NF-κB), which, in its phosphorylated active form, regulates the expression of pro-inflammatory and pro-apoptotic genes [22,23,24]. We observed a reduced expression of the pro-inflammatory cytokines IL-1ß and TNF-α, and found a decreased expression of the pro-apoptotic protein Bax and an increased expression of the anti-apoptotic Bcl-2. The respective changes depended on the H_2_S concentrations and time intervals used, with the strongest effect at 80 ppm and a delay of 1.5 h. 

The underlying neuroprotective mechanism of inhaled H_2_S after acute neuronal damage has been previously analyzed in a few studies. Our results are in accordance with the findings reported by Wei et al. [16], who showed that inhaled H_2_S mediates cerebroprotective effects after induced cardiac arrest. They determined that H_2_S caused a reduction in NF-κB phosphorylation and decreased the release of the pro-inflammatory cytokines IL-6 and TNF-α. Similarly, Zhang et al. demonstrated the anti-apoptotic effects of exogenously supplied H_2_S in their study on traumatic brain injuries in mice. They found that H_2_S applied after injury reduced caspase-3 cleavage and increased Bcl-2 expression in injured brain tissue [25]. A neuroprotective effect of exogenous H_2_S was also demonstrated in a mouse model of induced Parkinson’s disease by the promotion of neurogenesis via Akt [26]. 

In the present study, we were able to show that inhaled H_2_S inhibits the NADPH oxidase 4 (NOX4) enzyme. The complex process by which IRI leads to the death of neuronal ganglion cells involves oxidative stress and pathogenesis caused by the formation of oxygen radicals [27,28]. A protective, anti-oxidative effect of H_2_S has previously been demonstrated in a variety of organs, including neuronal tissues such as the retina [29,30,31,32]. The production of reactive oxygen species by neutrophil granulocytes occurs via NOX4 activity. For example, Wang and colleagues demonstrated that exogenously supplied H_2_S significantly reduced cerebral NOX4 expression in a mouse model of middle cerebral artery occlusion [33].

Endogenic hydrogen sulfide is produced in many tissues and seems to play an important role in physiological and pathophysiological processes [34,35]. In the retina, H_2_S is predominantly produced by CBS and cystathionine γ-lyase (CSE), which are localized in the ganglion cell layer [36]. The function of endogenous CBS in the retina is unclear at present. However, some evidence supports a role for endogenously produced H_2_S in ischemic brain injury and that elevated H_2_S levels contribute to poor outcomes [37]. We demonstrated that the inhalation of 120 ppm H_2_S strongly reduced the expression of CBS after IRI. The lack of a protective effect strengthens the hypothesis that a certain toxic component, such as the inhibition of the respiratory chain, counteracts the H_2_S effects—at least in a minor way.

## 4. Materials and Methods

### 4.1. Animals

We used adult male and female Sprague–Dawley rats (1:1 male-to-female ratio, body weights of 280–350 g, Charles River, Sulzfeld, Germany) for these experiments. The animals were fed a standard diet ad libitum and kept on a light/dark cycle of 12 h of each. All interventions performed were in accordance with the ARRIVE guidelines for the use of animals in research and were approved a priori by the Committee for Animal Care of the University of Freiburg (approval number: 35-9185.81/G-16/46). All types of operations and manipulations were performed as previously described [38,39,40]. The number of animals for RGC quantification and molecular analysis was *n* = 8 per group. For the analysis of the mRNA and protein expression and immunohistology, the retinal tissue was collected 24 h after H_2_S inhalation.

### 4.2. Retrograde Labeling of RGC

Sprague–Dawley rats were anesthetized with isoflurane and placed in a stereotactic apparatus (Stoelting, Kiel, Germany). The skin over the skull was incised and retracted. For drilling three holes on each side of the Sagittal suture, the Lambda and Bregma sutures served as landmarks. Then, a total amount of 7.8 µL of fluorogold (FG) (Fluorochrome, Denver, CO, USA)—dissolved in DMSO/PBS—was injected through the drilling holes into both superior colliculi. To ensure the adequate retrograde transport of FG into the perikarya of the RGC, further experimental procedures were conducted seven days later.

### 4.3. Retinal Ischemia/Reperfusion Injury and H_2_S Treatment

After randomization, rats received intraperitoneal sedation. Next, the anterior chamber of the left eye was cannulated with a 30-gauge needle connected to a reservoir containing 0.9% NaCl. The intraocular pressure was raised to 120 mm Hg due to the hydrostatic pressure of the water column. The immediate onset of ocular ischemia was confirmed by the microscopical detection of interrupted retinal blood flow. After 60 min, reperfusion was initiated by the removal of the needle tip. Failure to immediately restore retinal perfusion at the end of ischemia resulted in the exclusion of the animal, as did lens injury since it prevents RGC death and promotes axonal regeneration [41]. To evaluate the neuroprotective effect of inhaled H_2_S, the animals were randomized to receive either room air or H_2_S at concentrations of 40, 80, or 120 ppm in 21% oxygen and the appropriate amount of nitrogen (Air Liquide, Kornwestheim, Germany) for 60 min. Therefore, animals were placed in an airtight chamber (the dimensions of the chamber were 20 × 35 × 27 cm; the flow rate was 8 L/min initially for 10 min, then reduced to 2 L/min). Treatment with hydrogen sulfide was initiated either immediately after reperfusion or with a delay of 1.5 or 3 h.

### 4.4. RGC Quantification

For RGC quantification, the animals were sacrificed by CO_2_ inhalation 7 days after ischemia. Immediately removed retinal tissue was placed in ice-cold Hank’s balanced salt solution and processed further for whole-mount preparation. The retinas were carefully placed on a nitrocellulose membrane with the ganglion cell layer (GCL) on top. Subsequently, remnants of the vitreous body were removed and the retinae were fixed in 4% paraformaldehyde for 1 h and embedded in mounting medium (Vectashield; Axxora, Loerrach, Germany). The densities of FG-positive RGC were determined in a blinded fashion using a fluorescence microscope (AxioImager; Carl Zeiss, Jena, Germany) and the appropriate bandpass emission filter (FG: excitation/emission, 331/418 nm), as described previously [38,39,42]. Briefly, we photographed 3 standard rectangular areas (0.200 mm × 0.200 mm = 0.04 mm^2^) at 1, 2, and 3 mm from the optic disc in the central regions of each retinal quadrant. Thus, we evaluated an area of 0.48 mm^2^ per retina. To calculate the average RGC density in cells/mm^2^, we multiplied the number of cells analyzed/0.04 mm^2^ by 25. Secondary fluorogold-stained-activated microglial cells (AMC) after RGC phagocytosis were identified according to morphologic criteria and excluded from the calculation. All data are presented as the mean RGC densities (cells/mm^2^) ± SD. 

### 4.5. Real-Time Polymerase Chain Reaction

From retinal tissue harvested 24 h after ischemia, the total RNA from one-quarter of the retina was extracted using a column purification-based kit (RNeasy Micro Kit, Qiagen, Hilden, Germany) according to the manufacturer’s instructions. Reverse transcription was performed with 50 ng of total RNA using random primers (High Capacity cDNA Reverse Transcription Kit, Thermo Fisher Scientific, Waltham, MA, USA). Real-time polymerase chain reactions (RT-PCR) were done with a TaqMan^®^ probe-based detection kit (TaqMan^®^ PCR Universal Master Mix, Thermo Fisher Scientific, Waltham, MA, USA). The following primers were used: Bax #Rn01480161_g1, BCL-2 #Rn99999125_m1, IL-1ß #Rn00580432_m1, TNF-α #Rn01525859_g1, NOX4 #Rn00585380_m1, CBS #Rn00650948_m1 (all from Thermo Fisher Scientific, Waltham, MA, USA). The PCR assays were then performed on an RT-PCR System (StepOnePlus^®^, Thermo Fisher Scientific, Waltham, MA, USA) with the following cycling conditions: 95 °C for 10 min, 40 cycles of 95 °C for 10 s, and 60 °C for 1 min. Reaction specificity was confirmed by running the appropriate negative controls. The cycle threshold (CT) values for each gene of interest were normalized to the corresponding CT values for glyceraldehyde 3-phosphate dehydrogenase (GAPDH) (ΔCT). The relative gene expression in IR injured retinal tissue, either with the inhalation of hydrogen sulfide or room air, was calculated in relation to the corresponding gene expression in the non-injured retinal tissue of each individual animal (ΔΔCT).

### 4.6. Immunohistochemical Staining

For this analysis, eyes were enucleated 24 h after ischemia, immediately placed in 4% paraformaldehyde for 1 h at 4 °C. After washing in Dulbecco’s phosphate-buffered saline (D-PBS) before and after post-fixation in 20% sucrose for 4 h at room temperature, the eyes were embedded in Tissue-Tek (Sakura-Finetek, Torrance, CA, USA) and frozen in liquid nitrogen. Frozen sections (10 µm) were cut through the middle third of the eye and collected on gelatinized slides. Immunohistochemistry was performed according to standardized protocols [38]. A polyclonal Rabbit anti-TNF-alpha antibody was used (#ab6671, dilution 1:500, Abcam, Cambridge, UK) and detected with a corresponding fluorophore-conjugated secondary antibody (Alexa Fluor 647, mouse anti-rabbit, dilution 1:1000, Jackson ImmunoResearch Europe, Newmarket, UK). Retinal cell nuclei were stained by adding 4′,6-diamino-2-phenylindole dihydrochloride hydrate (DAPI; Sigma) to Mowiol mounting medium. The slides were examined under a fluorescence microscope (Axiophot, Carl Zeiss, Jena, Germany).

### 4.7. ELISA

Serum samples were analyzed using a commercial kit for TNF-α (#RTA00, R&D Systems, Minneapolis, MN, USA) according to the manufacturer´s instructions. OD was analyzed at 450 nm on a microplate reader (Tecan^®^, Männedorf, Switzerland). 

### 4.8. Western Blot Analysis

After 24 h of ischemia, retinal tissue was collected for protein expression analysis. The total protein from three-quarters of the retina was extracted and processed for Western blot, as previously described [42]. The membranes were blocked with 5% skim milk in Tween20/PBS and incubated in the recommended dilution of protein-specific antibody (p-NF-κB#3033, p-Akt#9271, Cell Signaling Technology, Danvers, MA, USA) overnight at 4 °C. After incubation with a horseradish peroxidase-conjugated anti-rabbit secondary antibody (GE Healthcare, Freiburg, Germany), the proteins were visualized using enhanced chemiluminescence Western blotting detection reagent (Western Lightning plus enhanced chemiluminescence, #NEL104001EA, PerkinElmer, Waltham, MA, USA) following the manufacturer´s instructions. For normalization, the blots were re-probed with total NF-κB (#8242, Cell Signaling) and total Akt (#9272, Cell Signaling). Relative changes in protein expression in IRI retinas after either hydrogen sulfide or room air inhalation were calculated relative to corresponding nonischemic retinas. Recordings and densitometric analysis were performed using Fusion Fx^®^ (Vilber, Collegién, France).

### 4.9. Statistical Analysis 

A computerized statistics program (SigmaPlot Version 11.0, Systat Software Inc., San Jose, CA, USA) was used to evaluate the data. After checking the normal distribution of the data, the results were presented as mean values (SD). Between-group comparisons were performed with one-way ANOVA using the post hoc Holm–Sidak test and Kruskal–Wallis test for data with missing normal distribution. Statistical significance was assumed at *p* < 0.05. 

## 5. Conclusions

We demonstrated that inhaled hydrogen sulfide protects retinal ganglion cells against ischemia–reperfusion injury. The effect depends on the time of application as well as on the administered dose. H_2_S mediates part of its effect via anti-oxidative, anti-inflammatory, and anti-apoptotic properties, while its molecular mechanism may be strengthened by an NF-κB and Akt dependent pathway.

## Data Availability

All data generated or analyzed in this study are included in this published article.
